# The Precarious Space for Mourning: Sick Leave as an Ambiguous Topic in Bereaved Parents’ Accounts of the Return to Everyday Life After Reproductive Loss

**DOI:** 10.1007/s11013-021-09728-3

**Published:** 2021-06-16

**Authors:** Ellen Kristvik

**Affiliations:** Health Services Research Unit, Akershus University Hospital (Ahus), P.O.Box 10005, 1478 Lørenskog, Norway

**Keywords:** Stillbirth, Bereavement, Sick leave, Parental rights, Norway

## Abstract

This article addresses conflicting concerns related to space for mourning in Norway. It draws on material from qualitative interviews with bereaved parents who have lost a child in stillbirth. Space for mourning, and the need for sick leave, arose as a crucial concern and complex issue in these interviews. Although initiatives have been developed to introduce grief as a valid category in diagnostic repertoires, it is not a legitimate basis for sick leave in the acute phase. Common alternatives have been referrals to psychic instability or depression. Both variations represent a medicalization of the normal with implications that need to be addressed, and which this article discusses from the bereaved parents’ point of view. Extended parental leave, and the introduction of grief allowance, are possible alternatives for the provision of space in normal but demanding times of grief. Despite not yet part of the repertoire for gatekeepers in the Norwegian welfare state, they are part of the public discourse. Besides a crucial acknowledgment of the grief of the parents, these options also represent possibilities for preventing a pathologization of what is a normal rite of passage.

## Introduction

I have never been happier than I was before this happened, you know. Everything was fine, and it was so close to the birth. Everything was better than it had ever been. Then all of a sudden, it is worse than it has ever been, and that distance!

These words come from Gerd, one of the bereaved parents I met after the loss of a child in stillbirth. Gerd, who works as a secretary, is married to Gustav, a sales agent in an international firm. After years of trying to get pregnant, they were at long last expecting a child, but their daughter died 1 month before she was due. When I meet them 10 weeks afterwards, the grief is still dominating their lives. Gerd is still on full-time sick leave, not knowing when she will be able to return to work. Gustav is not on sick leave, but he spends practically all of his time at home with his wife. Most days, they are together from morning until the evening. Gerd and Gustav both describe an all-consuming feeling of exhaustion. As Gustav says:

There is only chaos in my head. The office is not a place for me to be right now […] We are exhausted! Even on days when we hardly do anything. […] I went to the office one day, and that day I fell asleep sitting in my chair. I woke up and was awake for an hour or so before falling asleep again. I mean: Then you are weary! You shouldn’t sit and sleep.

Gerd expresses a struggle to manage everyday life in a way she does not expect other people to understand. Relating to others is a strain:

I am sure people may think: Is it really that big a thing? But there is nothing else in my whole career that has kept me away from work for so long. […] There is no joy in anything. Never in my whole life have I experienced losing my spark like this. It feels like not wanting to relate to others for the rest of my life.

Grief is a universal, deeply personal, and pervasive experience. Different kinds of assistance and provisions are offered to those who have suffered a loss through leave-taking rituals and temporary exemptions from tasks and duties. Space for mourning is also included in van Gennep’s description of funerals as rites of passage (van Gennep 1960:146ff). The transitional period for the bereaved, which follows rites of separation and ends through rites of reintegration into society, is often highly regulated. Special clothing and other external signs of mourning may elicit sympathy for the mourners as well as indicate respect for the dead. They may provide protection from unwanted encroachment on private grief and mark a period of suspension of social life (Taylor 1983). The practice of wearing black clothes, sometimes changed to black arm ribbons, has also been part of the Norwegian tradition but is now mostly limited to funeral attire etiquette (Aagedal 1994:79). Such signs, rules, and regulations are also indicators of social status and mourners’ relationship to the deceased.

In Norway, as in other countries in this part of the world, much has happened over the last few decades with regard to the status of a stillborn child. Losing a child in stillbirth is now more recognized as a loss in its own right and a legitimate cause for mourning. In accordance with the first tasks of mourning outlined by Worden, defined as accepting the loss and confronting the pain of grief (Worden 2009), midwives and nurses are expected to provide bereaved parents with opportunities to spend time with the dead child (Scott, Henley, and Kohner 2016; Layne 2003; Peelen 2009; Kristvik 2014). After departure from the hospital, however, guidelines are scarcer. Professional practices vary concerning the provision of the space required for continuing the tasks of mourning, adjusting to the new situation, and finding an enduring connection with the deceased.^1^ In the current Norwegian context, sick leave is one of the main ways this space is provided.

What are the roles and implications of sick leave in connection with loss and bereavement? Are grief and mourning to be regarded as an illness or as a rite of passage and normal life event? This question, posed by Maurice Eisenbruch three decades ago, is still relevant in studies with a cross-cultural perspective (Eisenbruch 1984).

In this article, I explore the question of a possible medicalization of a non-medical event in relation to grief that follows the experience of losing a child in stillbirth. I discuss this with a focus on temporary exemptions from tasks and duties, i.e., Norwegian rules and regulations for absence from work after prenatal loss. I address dilemmas and contradictions these systems imply and how they are responded to by the bereaved. My discussion is based on interview material with bereaved parents from my research project on parting with children who die before being known.

## Systems of Leave Relevant to Reproductive Loss in the Norwegian Welfare System

The two main systems of leave that are applicable for granting absence from work after prenatal loss in Norway are maternity leave and sick leave, both of which are provided by the state and financed by taxes.

Sick leave was introduced to ensure the income of employees in times of impairment. The current system is part of the larger project of the welfare state, which gradually replaced previous charity arrangements like poor relief funds and the dole.^2^ With this system, full compensation is guaranteed from day one. The employer is economically responsible for the first 2 weeks, after which the state takes over, with one hundred percent coverage of the salary up to a maximum of 12 months. After that, the compensation is reduced by one third, but the employee still has the right to keep his or her job. Sickness is not a legitimate cause for dismissal in Norway.

A precondition for being entitled to these benefits is permanent, regular employment. Weaker forms of appointments still imply a right to sick leave, but the job security is not as definite, and the protection against untimely termination of job contracts because of sickness absence is less dependable. On the irregular market, the question of job security, and paid leave of any kind, is unreliable to a much higher degree.

The sick leave system is based on trust. The employee’s self-report suffices for the first 3 days. A doctor’s certificate is required for longer absences. The physician’s assertion of the patient’s impairment has customarily not been questioned by other authorities. Costly as it is, however, the sick leave system has been increasingly contested over the years. Reduction in the benefits, e.g., through the introduction of unpaid waiting days (*karensdager*) and decreasing the compensation have been repeatedly launched as political proposals, opposed and rejected by the labor movement and the trade unions.

Attempts to alter the basic rules and regulations have so far been unsuccessful. However, the authority of the prescribing doctor is not as sovereign as before. Doctors employed by the social security institution (NAV) may question and overrule the prescribing doctors’ decisions. This is specially done by a demand for more standardization: a prescription practice where the duration of sick leave corresponds with the diagnosis in a precise and predictable way (Molander, Grimen, and Eriksen 2012). Professional discretion, essential as it has been to the practice of medicine, is thus undermined. Experienced doctors with more authority and awareness of the internal logic of the system have a relatively stronger position, with greater negotiation power in relation to this pressure. Patients with less experienced doctors may be correspondingly vulnerable.

Sick leave in Norway is, thus, granted on the basis of an international system of diagnosis that is increasingly standardized and subject to control. Acute grief is not part of this diagnostic system, although it is widely recognized that grief affects the health and work capacity of the bereaved. Anxiety, sleep disorders, concentration problems, poor memory, intolerance for stress, exhaustion, and fear of emotional breakdown are frequently reported health issues after a significant loss (Hazen 2006; Stroebe, Schut, and Stroebe 2007). In the system of diagnosis currently applied in Norway, however, acute grief is not only not a diagnosis in its own right; it is also an explicit exclusion criterion for sick leave based on symptoms that are otherwise used as a basis for depression (Lamb, Pies, and Zisook 2010). Grief has recently been introduced as a separate diagnosis, but as complicated grief, i.e., symptoms that have persisted for more than 6 months (Kristensen 2013). The recent introduction of grief in the current diagnostic system is, thus, not applicable for the provision of space for mourning in the wake of a loss. As my material also confirms, bereaved parents are often on sick leave nevertheless, albeit with other diagnoses. That, however, is not without complications. I will return to some of those.

Parental leave began with 6 weeks for working mothers in 1930. Since then, it has gradually increased. In addition to the paid leave for the mother, a two-week quota specifically for the father was introduced in 1993. Initially mostly meant as a support for the mother in the first demanding days after birth, the emphasis has increasingly been on the significance of fatherhood, the father’s own right to spend time with the newborn, and the importance of the very beginning of life for establishing a close relationship between father and child. Accordingly, the quota for the father has expanded. The current system operates with a parental leave of a total of 47 weeks, which is divided into thirds: one third reserved for the mother, one third for the father, and one third to be shared between the parents as they decide.^3^

In the case of stillbirth, i.e., death after 22 weeks of gestational age,^4^ the mother is granted 6 weeks maternity leave by the state. Leave of absence is often prolonged beyond that, but as sick leave. There is no automatic provision of paternity leave, but 2 weeks’ leave is often provided by the employer, possibly extended as sick leave by the hospital or family doctor.

The rationality behind this inequality seems to be medically based: The body of the mother, prepared for receiving and nursing an infant, goes through an adjustment of a very physical kind. In addition to that, she has to deal with the mental impact of the loss, for which 6 weeks is seldom enough. While the father does not go through the same physical implications as the mother, he might be equally mentally affected by the lost dreams and expectations that a stillbirth implies. The present system, thus perceived, lacks an acknowledgment of the father’s loss. The recognition of fatherhood that permeates the current arrangement for parental leave is not reflected in the directives for leave after stillbirth.

Both sick leave and parental leave are rights of citizens of the welfare state, neither of which are universal or unconditional. For one thing, they are both reserved for productive citizens.^5^ Besides that, sick leave is a discretionary right, dependent on professionals’ judgments, while parental leave is supposed to be for everyone eligible to claim the status of a parent. That distinction was existential, and challenging, for some of the parents I met.

## Parting with Children Who Die Before Being Known: Outlines of a Research Project

The aim of this project was to understand bereaved parents’ unique perspectives on what they perceived to be helpful or harmful in the mourning process after losing a child in stillbirth. Based at a center for health services research, this was in accordance with the center’s overall objective to investigate the quality and efficacy of health care in Norway. The project sought to gain more knowledge about the implications of stillbirth for bereaved parents and the adequacy or deficiencies of the services offered to them. No external funding was granted for the project. As an employee of the research center, I carried out the project design, data collection, and processing of the material.

Sensitive as the subject is, acquiring acceptance from the regional ethical committee was a lengthy process that took more than a year. The procedure that was finally approved implied a delay of the formal consent for participation in the project. Initially, the parents only agreed to being contacted by the researcher through an open question about participation posed by a hospital chaplain. The letter of consent was signed at the time of the first interview.

This procedure necessarily implies a selective group of informants. Hospital chaplains are called for in all cases of stillbirth at the hospitals concerned. Being associated with a Christian community or creed is no precondition for the chaplains’ assistance. They act as counselors on a general basis and help to arrange memorial services across confessional borders. There were Muslims and Buddhists among my informants. However, this does not mean that the chaplains are reaching everyone. In addition to the possible confessional barrier, several situational factors may affect the willingness to meet the chaplain. Death before birth is often a deep tragedy. It can also be highly ambiguous and even a relief (Jutel 2006). That, however, is not the topic of this project.

The material was gathered between 2015 and 2019. Bereaved parents from three hospitals were recruited while still admitted in the hospital. Parents who were bereaved in connection with 29 stillbirths consented to being contacted by a researcher to be interviewed about their experiences. In ten cases, parents also gave permission for the researcher to be present in leave-taking events. When contacted by me, parents bereaved in connection with 25 stillbirths agreed to be interviewed. Eighteen couples were interviewed with both of them present. Most of the informants were interviewed twice. The timing of the interviews was carried out with flexibility. In most cases, the first interview took place 2–6 months after the loss and the second at least 1 year later. The interview duration was between 1 and 3 h. Whenever possible and acceptable to the informants, the interviews were conducted in their own homes.

The interviews were predominantly narrative, with open-ended questions. While my initial interest as a researcher was in leave-taking events and the narrow window of opportunity soon after birth, the course of the interview largely depended on what appeared as compelling concerns for the people I met. The elaboration of different parts of the process, thus, varied among different informants. Covering the resumption of the tasks of everyday life was part of the initial design. Sick leave, however, was not a specific issue to start with but emerged as a crucial question for many of the informants.

The material highlights bereaved parents’ perspectives on a range of issues pertaining to their experience of the mourning process. This will be further elaborated in other publications, each of which will be focused on a particular group of professional helpers: nurses and midwives, hospital doctors, hospital chaplains, and psychotherapists. In this article, I focus on bereaved parents’ accounts of connections between their grief and their work and how the question of sick leave was raised by the people I met. The professional group most relevant in this context is general practitioners.

## People I Met: Parents Bereaved After the Loss of a Child in Stillbirth

### Physical Exhaustion and Mental Chaos

The weariness and distress described by the couple at the beginning of this paper resonate with the way many of the other bereaved parents would characterize the state they were in.

Grief as an all-consuming experience and a total energy drain came up frequently in the parents’ accounts. Sometimes this developed into a sense of losing one’s mental capacity and a fear of going mad. This was the case with Lisbeth, whose daughter died in the 21st week of pregnancy. The couple had three children already, but this last one was still a very wanted child.

Lisbeth was active and composed in the first phase after the loss. She planned the funeral together with her family and continued to be her usual capable self for a while after that. However, when the hospital did not keep its word about offering them a consultation regarding the autopsy of the child, and a conflict came up at her workplace at the same time, Lisbeth broke down. When I met her, half a year after the loss, she had received therapeutic help and was able to talk about what had happened from a perspective:

Grief is so many things. I have understood that over time. When one thinks about grief, one thinks about being sad. But grief is not just being sad. It can turn out as restlessness. I struggled with remembering; I struggled with concentrating. My thoughts were very chaotic, constantly going on loops in one big mess.

[…] I did not know that, but it can go as far as wondering if one is about to turn mad, you know! You think that you are about to topple over. Everything is just a big chaos. I have thought about that: It is so easy to recognize when seeing it in others, but you don’t see it in yourself. It is impossible to realize what is going on.

Lisbeth describes a delayed reaction. Hers is a story of a bereaved mother who initially seemed to cope very well after the loss but broke down after a while. When that happened, she needed professional help to sort out her feelings and recognize the emotional turmoil for what it was. In Lisbeth’s case, this mental collapse happened after 5 months. Because of a pilot project in her vicinity, more open for admissions than the regular services, she was given access to therapy. Delayed grief reactions, or unresolved grief, may manifest years after the loss. When 6 months have passed, it qualifies as a diagnosis, with appurtenant rights to therapeutic assistance.

### Social Vulnerability

Several of the parents also described vulnerability in social situations in the time that followed the loss. Gerd describes a self-centeredness, which she herself thinks of as odd:

It is pretty extreme, I think. In the beginning, especially, I very much felt like talking to people, but I talked only about what had happened, was only centered on myself. And when people began talking about different things, I was not able to talk about anything else – nothing at all! I only got angry and irritated. It was just terrible if people came here and began talking about something else, joking and talking nonsense. I just wanted to ask them to leave: Don’t you understand what has happened?! Do you understand nothing at all?!

Gerd’s partner, Gustav, was granted 2 weeks of paternity leave by his employer, the same amount of time as he would be entitled to if the child had been born alive. After that, as a leader in a private firm, he was able to make an agreement with his employer that allowed him to work from home and make his own choices about what tasks to prioritize. He was exempted from most regular contact with clients and customers and traveled much less than he normally did. He still attended certain meetings at the local sales office, which he judged to be crucial for keeping track of important developments and followed these up from home.

Gerd informed her colleagues about the stillbirth during a spontaneous visit to her workplace shortly after the loss. After the 6 weeks of maternity leave, she dropped by her office to hand in her sick leave papers. She had been hesitant about doing it that way but had been encouraged by friends who assured her it was a smart thing to do. She arrived unannounced, broke into tears as soon as she arrived, but ended up staying for 3 h. Even though this was well received, she is later ambivalent about having done this. She perceives herself as a private person and does not have any particularly close interpersonal relations at her workplace. Afterwards, she is worried that she might have exposed herself too much: “I felt that I had turned my soul inside-out to them then and there.”

Feelings of vulnerability and unbalance in contact with others, as Gerd talked about, resonated with much of what others also described. Breaking the news about the stillbirth to people who did not already know, and perhaps were eagerly asking about the baby, was mentioned by many as a particularly painful thing to do. One of the bereaved mothers I talked with brought up the traditional arm ribbons as a practice she wished could have continued, a way of making others understand her state of mourning without her having to explain it.

### Work as Relief

Few of my informants returned straight to work after the prescribed leave of absence. According to his own wish, though, David returned to work after 2 weeks. He asked his boss to inform his colleagues and told him that he did not want extra attention and wanted to continue as before. For him, that was helpful: “That was my way of handling things. Just to basically work. I don’t know if that’s the best way, but it has helped. And still does.”

Most of the bereaved fathers I met requested and were granted, more time off than this. But David’s rather fast return to work could seem to confirm a common perception or stereotype about gender differences with regard to grief: Men are supposedly less affectively expressive than women, more prone to problem solving and less prepared to confront and work through the pain (Martin and Doka 2000). In my material, more men than women talked about restlessness and a search for distraction as a way to deal with the pain. During the interviews, however, many of the fathers were just as emotional as their partners and just as prone to tears when talking about their loss.

Work can be a welcome distraction from pain. That it also can have other qualities crucial to the space for mourning is shown in the story of Katrine, who also went back to work by her own choice soon after the loss. Katrine is a single woman who works as a nurse at an intensive ward for premature children. She was 11 weeks pregnant with her first child when she was told that the condition of the child she carried was incompatible with life, and she was advised to take an abortion. Katrine did what she could to test that information and explore the possibilities for the child to live, but after 10 weeks of intense investigations, she accepted this, and the birth was induced when he was 21 weeks. When I meet her, 18 months after the loss, she has given birth to another child, 3 months old at the time. Katrine’s colleagues understand that even though she is delighted about her son, she still needs to talk about the baby she lost. Knowing about that support was important for her prompt return to work, 4 weeks after the loss.

When he was born, I just wanted to go back to work. Staying at home was not good for me then. By that time, I had processed so much – I had been thinking so much – the rest had to be kind of processed on the way.

As a nurse in the premature ward, Katrine had been leading bereavement groups for parents who had lost children soon after birth.

K: You are quite alone in your grief after losing a child in stillbirth. For nobody knows those children. Nobody has experienced anything with them. So, for many people, it is difficult to relate to that grief. Life goes on pretty fast. I have seen how huge the need is for being recognized and listened to in a way that resonates with others. And I saw the same thing in my circle of friends – they disappeared quickly – those out there. The attention to what I had gone through disappeared very fast. I thought I was prepared for that, but even so, it took me by surprise. I can understand it, but feeling it is different.

E: Was that part of why it was good to return to work?

K: Yes, that is right! Because at work, people talked about it all the time. It is kind of strange, I guess, but it was completely natural for people to talk about my first son. And they kept talking about him when I got pregnant again, too. My friends and family did not do that. They kept completely silent about him. So, it (being with colleagues) was simply wonderful.

Katrine’s story, like David’s, shows that returning to work soon after the birth of the stillborn child can also be of help and not necessarily an impediment. An important part of Katrine’s story, however, is the 10 weeks she spent before giving birth, aware of the serious impairment of her child, though she did what she could to find out if he could possibly be helped to survive. It was also a time in which she could prepare for what was ahead of her. When the child was born, the mourning process had thus already begun.

Her work in the premature ward, where many children were fatally ill and frequently died, also meant an extraordinary working environment. Her colleagues related actively and emphatically to what had happened, to a greater extent than her family and friends.

### The Limitations of the Benefit: Sick Leave as a Risky Boon

David and Katrine returned to work early, by their own choice. The sense of choice was not there for Anna, a Bulgarian immigrant to Norway who works in a canteen. In accordance with her own choice, we meet in a café, without her husband.

Anna has a teenager from a previous relationship but lost the child she and her current husband were expecting at 23 weeks. Anna was on part-time sick leave because of intermittent pain during the pregnancy. Because of the timing of the loss, she was only granted 2 weeks of absence before she was expected to return to work. Anna did not feel ready to return but was worried about her position in the canteen, which was acquired through an employment agency. When I meet her, 2 months afterwards, she still has a pain problem and feels depressed. She feels unfit for work, but her fear of losing her job prevents her from asking for sick leave.

Anna’s case illustrates the limitations of the sick leave system. With a doctor’s prescription, she has the right to be absent from work. But by further utilizing that right, after the time, she spent on sick leave during pregnancy, she risks losing her job altogether. As a substitute worker, she does not have the protection from dismissal because of sickness that regular employment would imply.

### Sick Leave as Humiliation

I met Ragna 8 months after the loss of her second child, who died 4 days before she was due to be born. We meet in her apartment in a suburb of Oslo, to which she has recently moved after being separated from her husband. The death of their child was a huge strain on the relationship. Their eldest daughter divides her time between her parents, and is with her father when I visit. While both parents are present for most of the interviews, this is one of those I conduct with the mother only. Ragna and I sit alone on her small balcony, overlooking the woods that surround the town.

Ragna works as an ambulance driver. Thus, she is used to dealing with emergencies and critical situations. And thus, it was her own colleagues who came to fetch her when she suddenly began bleeding heavily at the end of a pregnancy that had been free of complications until then.

She had never been on sick leave before. When the maternity leave she was entitled to was over, she did not feel ready to return to work. But she was not prepared to ask for sick leave either. She did not feel sick. She was just not physically or mentally finished with what she had been going through. Neither was her boss willing to allow her to return to her normal tasks and responsibilities.

I really did not want to be on sick leave. That felt like a defeat for me. I really think that prolonged maternity leave should be an option. […] There are many physical things to go through before being ready for work. And then one needs time to patch up mentally – not because of being so sad all the time, but because of being so exhausted. This grief drains your energy.

Going on sick leave felt humiliating to Ragna, even though she wanted and needed more time off. What she brings up as a preferable option is prolonged maternity leave. That would give her space for healing and mourning as an evident right not to be questioned or assessed by someone else.

Another parent who strongly opposed the idea of sick leave instead of parental leave in the wake of his loss was a father. I met Fredrik and his wife Frida, who both work for private companies, 5 months after the loss. Struggling with insomnia and concentration problems, Frida had been on full-time sick leave for 1 month after her maternity leave. After that, she was permitted to work-from-home part time, according to her capacity.

We met in the evening, after work and dinner. Photos of their son, taken at the hospital, have a prominent place in the room where we sit. A framed picture of his initials, drawn large in clear colors, hangs on the wall in front of me, just behind the couple.

At the time of the interview, Fredrik had resumed work 100 percent. Reaching that point, however, had been a frustrating process. He had initially stayed at home for 2 weeks before trying to return to full-time work without consulting the doctor. As he too suffered from acute insomnia, he realized that he was unable to attend properly to his tasks at work, and when consulting the doctor, he was granted a 50 percent leave for 1 month. At that point, Fredrik’s employer asked him to procure a sick leave certificate for the initial 2 weeks of absence. To Fredrik, who had thought of this as regular paternity leave, this felt like an insult and a lack of recognition of his loss. Defending his right to an initial 2 weeks defined as paternity leave, instead of just sick leave, became a crucial endeavor and something he felt he owed to the child.

I have this one point of dissatisfaction regarding my workplace. I was told in advance that I would get two weeks of leave after the birth. All fathers have a right to two weeks of unpaid leave after birth. But in most workplaces, two weeks of paid leave is granted after birth. But when talking to my boss, I was told that no, the best thing would be for me to get a sick leave certificate from day one […] I got that message when we were still at the hospital. But coming back, I tried to find out a bit more, and I realized that the definition of birth applies from 26 weeks onwards – whether the child is dead or alive. That is what is understood by birth. So, I was puzzled: Why did I get that answer? I realized that they did not have things in order regarding that. So, I have tried to make them understand the situation: We have given birth to a child, and why should I not have the same kind of rights as the other employees? But they have not been able to grasp that as yet.

Fredrik was informed that the company had arrived at a policy decision about recommending that sick leave be applied for, but the company would pay if the hospital or the family doctor did not grant it. 2 weeks of paid leave would thus be guaranteed. But this was not acceptable for Fredrik. Though he realized that his case was settled, he was concerned about those coming after him:

This is very important to me. Both as a principle, and because I want the company I work for to recognize my child.

Like Ragna, Fredrik wants and commends parental leave instead of sick leave. In his case, this is an outright demand. To him, the denial of paternity leave after the loss of a child in stillbirth is a plain mistake, not consistent with the overall system of rights to which he belongs. And there is much at stake. Instead of his existence as a father being a precondition giving access to parental rights, the right he claims becomes a precondition for being acknowledged as a parent. By claiming his right to parental leave, he constitutes himself as a father. Thereby, he also constitutes the personhood of his child.

Fredrik launches a fight in the capacity of being a productive citizen, an employee. It is as such that he can fight for being paid while staying at home after the loss of his child. It is not a fight for money. Economic compensation for his absence from work is ensured for him. His compelling concern is what this is to be called and the identity thereby implied. As such, it might be compared with the movement behind the Missing Angels Bill in the USA, which fights for the right to a birth certificate for the stillborn (http://missfoundation.org), instead of the death certificate that is ordinarily issued. This fight also constitutes the women as mothers. According to Joanne Cacciatore, the founder of the movement, this is a difference between a reminder of a woman’s failure and an acknowledgment of the motherhood that remains real, also after the death of the child (Corriatore 2001).

## On the Issue of Medicalization and the Meaning of Sick Leave for Parents in Grief

Parents I met talked about the loss of their child as an overwhelming experience, which often had physical manifestations. The descriptions of their afflictions are very similar to the symptoms of depression. But while depression is a sickness in need of treatment, grief is a normal reaction in need of space and time. Naming it depression is a misleading labeling, and arguably an instance of pathologization.

Pathologization has been referred to as a narrower, more specific part of the broader issue of medicalization. The former has often been associated with the attribution of definitions and diagnosis, while the latter is meant to cover treatment and medical interventions (Conrad 2005:5). The two concepts are certainly related, though the possibility of both of them occurring independently has also been described (Sholl 2017). In this article I apply a definition of pathologization proposed by Svend Brinkman, i.e., “the process through which a painful or problematic phenomenon is turned into illness or disorder” (Brinkman 2019:2017). This can be a sheer matter of categorization, but it can also be more than that, when the labeling leads to conditions which contribute to the development of a pathological state.

Early works on the issue of medicalization were sharply focused on medical professionals, accusing doctors of imposing their power far beyond their legitimate domain (Friedson 1970; Zola 1972; Illich 1976). Retaining the argument about an ongoing medicalization of non-medical phenomena, later critics, like Peter Conrad, have maintained that the authority of doctors has been increasingly curtailed by the pharmaceutical industry, the market and systems of managed care (Conrad 2005). While commercialization has expanded and made an impact on health care services in Norway too, the increasing interference by the social security institution is a more direct imposition on the work of doctors, comparable to issues raised about managed care.

A focus on the system is also in accordance with accounts of the people I met. None of the bereaved parents brought up complaints about general practitioners in their interviews with me. But for some of them, the sick leave system itself did emerge as a cause of distress.

Part of the discourse on the issue of medicalization has concerned the internalization of the medical gaze (Foucault 2003), and a subsequent deprivation of people’s capacity to deal with normal but challenging parts of their lives (Illich 1976). This issue also been raised in the context of grief, and the introduction of grief as a diagnosis (e.g., Horwitz and Wakefield 2007; Frances 2013; Kofod 2017). I do not mean to reject that such an impingement on people’s self-perceptions, with curtailing effects on their agency, occurs. But after listening to the stories of the people I met, my concern about medicalization is not about that.

The self-presentations of the bereaved parents I met did not convey adaptations of a medical understanding of their grief as a pathological state. I did, however, meet with examples of ardent oppositions to the medicalization implied in a system based on diagnosis of depression and mental disturbance as the only option for leave of absence in the wake of a severe loss. Ragna, the ambulance driver, was one of those, though the nature of her workplace and a stern leader prevented her from a premature return to her duties. Ragna did not think of herself as sick but felt strongly affected by a tragic event and in need of time to deal with that. From her perspective, that could and should have been provided by extended parental leave.

The difference between parental leave and sick leave is not economic. The amount is not the same for everyone, but it is always a compensation of the income of the person concerned. The decisive distinction lies in the reason and rationale for the compensation, whether it is due to physical or mental impairment or the birth of a child, dead or alive.

Acute grief is not a pathological state. But it is an arduous condition that requires time, space, and flexibility. The lack of that space may increase the risk of developing a condition in need of professional assistance, and in that sense turning into a pathological condition. And when the initial space for mourning is made dependent on the medicalization implied in a doctor’s prescription with referral to a diagnosis of mental disorder, the risk is there for an outright rejection of that provision by the bereaved, even if space for mourning is a felt need.

Some of the parents I met were able to achieve space for mourning in an agreement with their employer, but most of them depended on a doctor’s prescription for a temporal exception from tasks and duties. Many appreciated the leave, thus, granted to them and did not seem particularly concerned about the ground on which it was granted. Disadvantageous future implications could still be there, as emerges in a survey conducted among bereaved people in Norway. Though most of the respondents claimed to have had no problems associated with that, a history of having been on sick leave also came up as a hindrance both in connection with applications for new jobs and for taking out an insurance policy (Christoffersen and Johannessen 2013:26).

Protection of privacy has a high standing in Norway. Information about sickness and health is regarded as sensitive material, and there is no obligation to provide such information to employers or insurance companies. Nor can anyone request information about a person’s sick leave or diagnosis without the permission of the person concerned. The possible consequences of withholding the information, however, may easily lead to a feeling of an untenable situation, and signing the provision of access to sensitive material is a common thing to do. One of the doctors I talked with said she brought this to her patients’ attention before applying one of the psychiatric diagnoses typically used in times of grief.

Fredrik’s objection to sick leave came from another place. His claim of the right to paternity leave was part of a fight for acknowledgment of the existence of his child. As such, it is also closely linked to the tasks of mourning and Fredrik’s possibilities for integrating the death of his child into the story of his life.

## Mobilizations for Alternatives to the Current System of Sick Leave

The fight that Fredrik, claiming his rights as a bereaved father, took up with his employer has been launched on a national level by bereaved parents’ interest organizations. It has been on the agenda of LUB (Norwegian SIDS and Stillbirth Society) for years. In other Scandinavian countries, arrangements for parental leave are already in place for bereaved fathers after stillbirths, more in line with the increasing recognition of fatherhood.

In cooperation with other national interest organizations for the bereaved and sister organizations in other Scandinavian countries, LUB has worked for alternatives to the current system of sick leave in times of grief. This includes the introduction of a grief statement (*sorgmelding*) instead of the current application of psychiatric diagnosis for the granting of sick leave in the wake of a loss, a proposal which has been supported by the national psychologists’ association as well as general practitioners. Their objection to the current practice is partly related to unwanted consequences for the individuals concerned, but it is also a concern about the accumulated picture: the concealment of grief, represented as sickness, on a societal level.

The question of grief allowance as an alternative to sick leave was also raised in LUB’s survey on grief and work referred to above (Christoffersen and Johannessen 2013), in which 88 percent of the respondents declared to be in favor of the system of grief allowance. When this survey was conducted, LUB sister organizations had been campaigning for a system of grief statements in Sweden, and an allowance of 10 days for bereaved parents had been introduced. The Swedish organizations had been aiming for more and continued campaigning for the allowance to be increased to 100 days.

Parallel to the Swedish campaign, LUB has worked for the introduction of grief money in Norway through knowledge acquisition, media presentations, and lobbying. Despite political promises to assess the issue, nothing has materialized so far. In Denmark, however, a new grief law was passed in June 2020. This happened after a campaign by a collaboration of organizations, fronted by the mother of a teenager who died from leukemia. According to the system of *borgerforslag* (citizen proposal), any citizen who can gather a sufficient number of signatures has the right to bring a given proposition up for discussion in the Danish parliament. The collection of the required 50,000 signatures led to the passing of a law ensuring the right to a grief allowance of 6 months for parents who have lost a child below 18 years.

The Danish achievement has inspired sister organizations in Norway and Sweden to renew their efforts. Organizations across Scandinavian borders are currently exchanging information and experiences to join forces for better grief support and the introduction of grief allowance in their respective countries.^6^ They argue that the total economic implications would be low, considering the relatively low number of parents concerned and the likelihood of them being on sick leave already.

## Concluding Remarks

The loss of a significant other is a universal but demanding and transformative life event, and the provision of space for mourning has been part of the ritual repertoire across a wide range of traditions. In Norway, the recognition of an extraordinary situation, comparable to the transitional period in van Gennep’s model of rites of passage, is granted by a medical expert through a medical diagnosis. Exceptions from regular duties and obligations, opening up a space for mourning, are rendered possible in the form of sick leave. From a medical point of view, that can be crucial in preventing what is a normal reaction from turning into a pathological state. It is, however, a medicalization of the basic need for space that implies a diagnostic labeling and is in that sense a pathologization in itself.

As indicated above, the medicalization of grief implies a level of arbitrariness. It means being subjected to control, not primarily by the prescribing doctors, but by the systems the doctors themselves are subjected to. It may backfire, with future repercussions beyond what the bereaved are immediately aware of, and it may also feel intensely humiliating at the time. An extension of parental leave after stillbirth, including paternity leave for the father, implies a recognition that may be crucial, especially at the onset of the bereavement process. With the possible supplement of a grief allowance, space for mourning is thus provided as another right from the welfare state, without pathologizing the receiver.

## Footnotes

1 Worden’s four tasks of mourning are distinctive from models of stages or phases of the mourning process, in its emphasis on the active subjectivity of the mourner. For more information, see Worden 2009:37–39. I see it as compatible with the dual process of mourning (Stroebe and Schut 2010) and the concept of continuing bonds (Klass et al. 1996; Root and Exline 2014).

2 For more info and analyses of the Norwegian welfare state, see Vike (2018).

3 A 50/50 division (6 months for mother and father both) has also been brought up as a political proposal. Women are divided on the issue: It is seen as an equality achievement as well as a deprivation of women’s rights.

4 This rule changed during my fieldwork. In accordance with the definition used in national statistics for births and deaths, the distinction between stillbirth (with rights to maternity leave) and miscarriage was moved from 26 to 22 weeks on March 1, 2019.

5 The right to birth money (*fødselspenger*) for students and unemployed people is limited to a one-time benefit after birth.

6 Trine Giving Kalstad, Chief advisor in LUB, personal communication, 2021.03.08.
