# Correction to: The Precarious Space for Mourning: Sick Leave as an Ambiguous Topic in Bereaved Parents’ Accounts of the Return to Everyday Life After Reproductive Loss

**DOI:** 10.1007/s11013-021-09733-6

**Published:** 2021-07-13

**Authors:** Ellen Kristvik

**Affiliations:** Health Services Research Unit, Akershus University Hospital (Ahus), P.O.Box 10005, 1478 Lørenskog, Norway

## Correction to: Cult Med Psychiatry 10.1007/s11013-021-09728-3

The original version of this article unfortunately contained an error. In the reference list the author name “Joanne Cacciatore” was incorrectly spelled as “Joanne Corriatore”.

The authors would like to correct the error with this erratum. The Correct reference is given below:

Cacciatore, Joanne 2001 MISSing Angels Bill Press Release https://www.missingangelsbill.org/index.php?option=com_content&view=article&id=51&Itemid=56. (Downloaded 2021.03.18).

The original article has been corrected.

